# Growth-suppressive activity of raloxifene on liver cancer cells by targeting IL-6/GP130 signaling

**DOI:** 10.18632/oncotarget.16898

**Published:** 2017-04-06

**Authors:** Yina Wang, Haiyan Ma, Chongqiang Zhao, Tianshu Liu, Dan Yan, David Jou, Huameng Li, Cuntai Zhang, Jiagao Lü, Chenglong Li, Jiayuh Lin, Sheng Li, Li Lin

**Affiliations:** ^1^ Division of Cardiology, Department of Internal Medicine, Tongji Hospital, Tongji Medical College, Huazhong University of Science and Technology, Wuhan, China; ^2^ Center for Childhood Cancer, The Research Institute at Nationwide Children's Hospital, Department of Pediatrics, College of Medicine, The Ohio State University, Columbus, OH, USA; ^3^ Division of Medicinal Chemistry and Pharmacognosy, College of Pharmacy, The Ohio State University, Columbus, OH, USA; ^4^ Department of Geriatrics, Tongji Hospital, Tongji Medical College, Huazhong University of Science and Technology, Wuhan, China

**Keywords:** raloxifene, GP130, STAT3, liver cancer

## Abstract

**Background:**

Interleukin-6 (IL-6) is a multifunctional cytokine, which is involved in the regulation of differentiation and growth of certain types of tumor cells. Constitutive activation of Signal Transducer and Activator of Transcription 3 (STAT3) induced by IL-6 is frequently detected in liver cancer and has emerged as a viable molecular target for liver cancer treatment. However, few inhibitors targeting up-streams of STAT3 are available for the therapy of liver cancer. We reported the discovery of EVISTA (Raloxifene HCl) as novel inhibitor of IL-6/GP130 protein-protein interactions (PPIs) using multiple ligand simultaneous docking (MLSD) and drug repositioning. The possible effect of Raloxifene in STAT3 signaling or liver cancer cells is still unclear.

**Results:**

Raloxifene inhibited the P-STAT3 stimulated by IL-6, but not the induction of STAT1 and STAT6 phosphorylation by IFN-γ, IFN-α, and IL-4. Raloxifene inhibited STAT3 phosphorylation and resulted in the induction apoptosis on human liver cancer cell-lines. Raloxifene inhibited the targets of STAT3, such as Bcl-2, Bcl-xl and survivin and cell viability, cell migration, and colony formation in liver cancer cells. Further, daily administration of Raloxifene suppressed the Hep-G2 tumor growth in mice *in vivo*.

**Materials and Methods:**

The inhibitory effect on STAT3 phosphorylation and activity as well as cell viability, migration, and colony forming ability by Raloxifene was examined in human liver cancer cells. Tumor growth was detected via mouse xenograft tumor mode.

**Conclusions:**

Our results suggest that Raloxifene is a potent IL-6/GP130 inhibitor and may be a chemoprevention agent for liver cancer by targeting persistent STAT3 signaling.

## INTRODUCTION

Hepatocellular carcinoma (HCC) is the third most common cause of cancer mortality worldwide and is the second cause of death from malignancy, following lung cancer. HCC accounts for more than 85% of all primary liver cancers, with a 5-year survival rate of 9% and a median survival time of less than 1 year. Recent data indicates that the mortality of HCC in China has been increasing, severely threatening the health and lives of people [1]. The large number of cases and poor survival rates under current therapies necessitates the search for novel target therapies for HCC.

As transcription factors, signal transducer and activator of transcription (STAT) proteins participate in relaying signals from cytokines and growth factors [2–4]. STAT3 has been found to contribute to oncogenesis including liver cancer [2, 5]. After activated, STAT3 translocates to nucleus, resulting in DNA binding and multiple oncogene transcription, which leads to cell proliferation, metastasis, angiogenesis, host immune evasion and resistance to apoptosis [6–9]. One of the cytokines that is able to induce STAT3 phosphorylation is interleukin-6 (IL-6). IL-6 binds to IL-6 receptor-α (IL-6Rα) to form a binary complex and then recruits GP130 to form the IL-6/IL-6Rα/GP130 heterotrimer. Further, homodimerization of the IL-6/IL-6Rα/GP130 heterotrimers occurs by interactions between IL-6 site III of one trimer and the D1 domain of GP130 of the other trimer, forming a hexamer [10–11]. The reciprocal homodimerization of the IL-6/IL-6Rα/GP130 trimers triggers a signaling cascade of phosphorylation of Janus kinases (JAKs) and a downstream effector STAT3, followed by reciprocal dimerization of the Tyr705-phosphorylated STAT3. Therefore, it is the key step for the formation of hexamer and cancer cell proliferation and migration that IL-6 binds to GP130. Thus, interference with STAT3 signaling pathway in cancer cells such as by antisense oligonucleotides and dominant-negative STAT3 mutants, has been shown to result in growth inhibition and the induction of apoptosis [12–14]. As the up-stream of STAT3, inhibition the binding of IL-6 and GP130 may also exhibit potent growth-suppressive activity in liver cancer cells. However, it will take a long time to develop new drug aiming at the binding of IL-6 and GP130. Thus, it is necessary to find new use of old drug.

As a microbial metabolite, Madindoline A (MDL-A) was reported to selectively inhibit GP130, resulting in STAT3 phosphorylation inhibition [15]. With the model of MDL-A, using a novel computational strategy for fragment-based drug design by combining multiple ligand simultaneous docking (MLSD) and drug repositioning, we demonstrated that Raloxifene exhibited a new function against IL-6/GP130 protein-protein interface in our previous study [16]. Although it is reported that Raloxifene inhibited constitutive STAT3 phosphorylation in pancreatic cancer cell and breast cancer cell-lines, whether it is efficient for Raloxifene to inhibit IL-6/GP130/STAT3 signaling in liver cancer cells and suppress tumor growth *in vitro* and *in vivo* is still unknown.

## RESULTS

### Inhibition of IL-6 mediated induction of STAT3 phosphorylation in Hep-3B liver cancer cell-line

**Figure 1:**
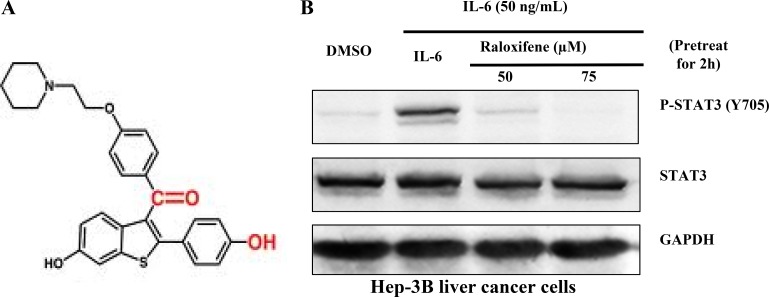
(**A**) The structure of Raloxifene. Raloxifene (marketed as Raloxifene by Eli Lilly and Company) is an oral-selective estrogen receptor modulator (SERM) that has estrogenic actions on bone and anti-estrogenic actions on the uterus and breast. (**B**) Raloxifene inhibits STAT3 phosphorylation induced by IL-6 in Hep-3B liver cancer cell-lines. With Raloxifene (50, 75 μM) pretreated for 2 h and IL-6 induced for 30 min, P-STAT3 was inhibited in a dose-dependent manner.

Previous studies have shown that Raloxifene had the potential to inhibit the combination of IL-6 and GP130 by multiple ligand simultaneous docking and drug repositioning technology according to the structure of MDL-A (Figure 1A). Studies have shown that Raloxifene selectively inhibits the phosphorylation of STAT3 induced by IL-6 in PANC-1 cancer cell-line [16]. To confirm whether Raloxifene has the potential of growth-suppressive in liver cancer, we first examined the effect of Raloxifene on constitutive STAT3 phosphorylation induced by IL-6 in Hep-3B liver cancer cells. With the pretreatment of Raloxifene for 2 hours and IL-6 added for another 30 min, we examined the expression of STAT3 phosphorylation on tyrosine 705 in Hep-3B. Our results showed that Raloxifene inhibited the phosphorylation of STAT3 induced by IL-6 in a dose-dependent manner and had no effect on the overall expression of STAT3 (Figure 1B).

### Effect of raloxifene on the phosphorylation of STAT3 induced by LIF and STAT1 and STAT6 induced by IFN-γ, IFN-α and IL-4

**Figure 2:**
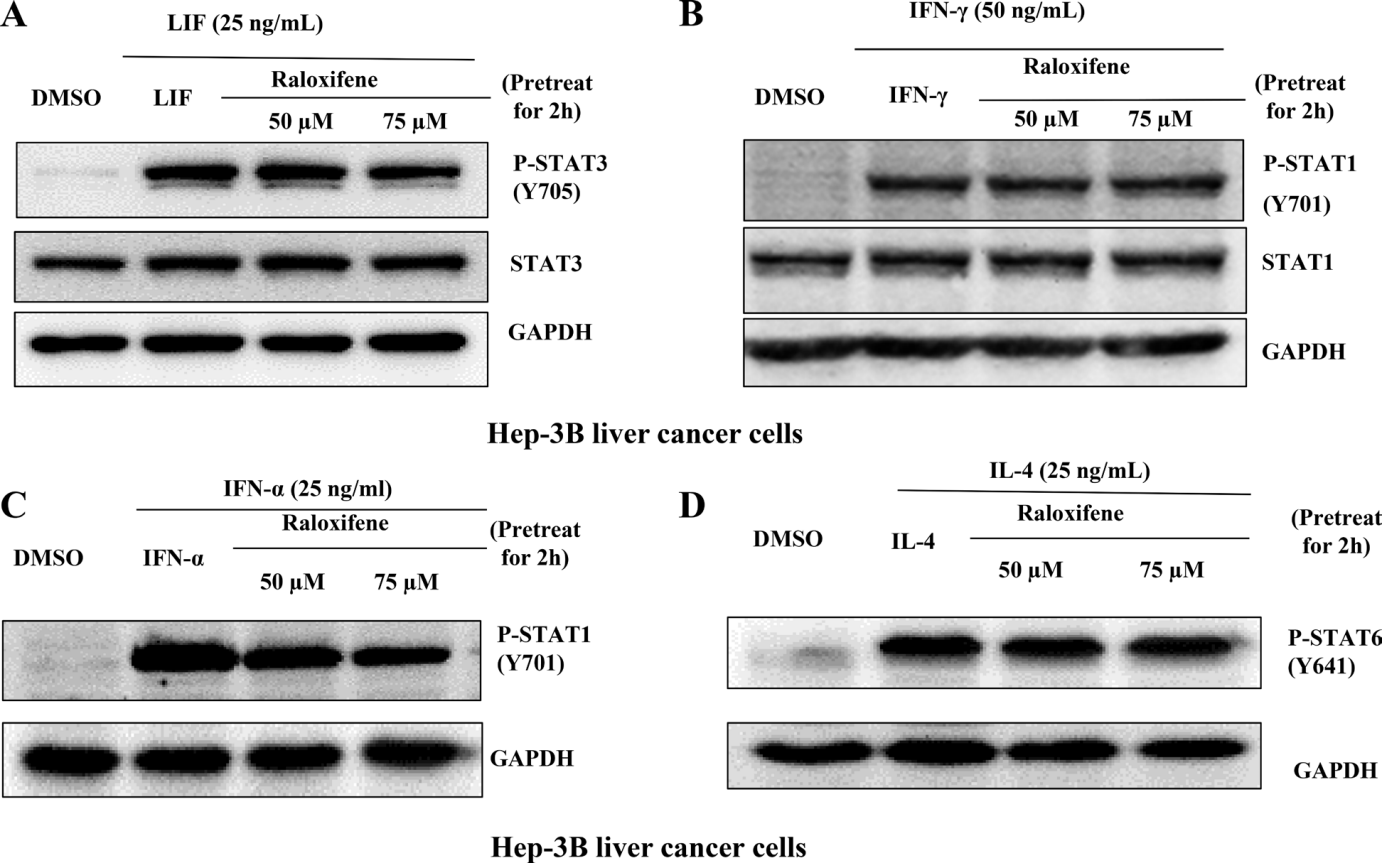
Raloxifene did not affect the phosphorylation of STAT3 on Tyr705 or other STATs induced by other cytokines Raloxifene did not inhibit the phosphorylation of STAT3 on Tyr705 induced by (**A**) LIF, did not inhibit the phosphorylation of STAT1 on Tyr701 induced by IFN-γ (**B**) and IFN-α (**C**), and did not inhibit STAT6 phosphorylation on Tyr641 induced by IL-4 (**D**) in Hep-3B liver cancer cells. With pretreated with Raloxifene for 2 h followed by cytokines inducing for another 30 min, western blot was used to evaluate the phosphorylation of STAT3, STAT1 and STAT6.

We also examined STAT3 phosphorylation on tyrosine 705 induced by LIF, STAT1 phosphorylation on tyrosine 701 induced by IFN-γ or IFN-α, and STAT6 on tyrosine 641 induced by IL-4 in Hep-3B liver caner cell-line. Our results showed that Raloxifene did not inhibit the phosphorylation of STAT3 induced by LIF (Figure 2A), or affect the level of STAT1 phosphorylation induced by IFN-γ (Figure 2B) and IFN-α (Figure 2C), and had no effect on STAT6 phosphorylation induced by IL-4 (Figure 2D) in Hep-3B liver cancer cells. The results indicated that Raloxifene specifically inhibited STAT3 phosphorylation induced by IL-6 significantly, but had no effect with other STATs induced by different cytokines.

### Effect of raloxifene on persistent STAT3 phosphorylation and the downstream target genes of STAT3 in liver cancer cell lines

**Figure 3:**
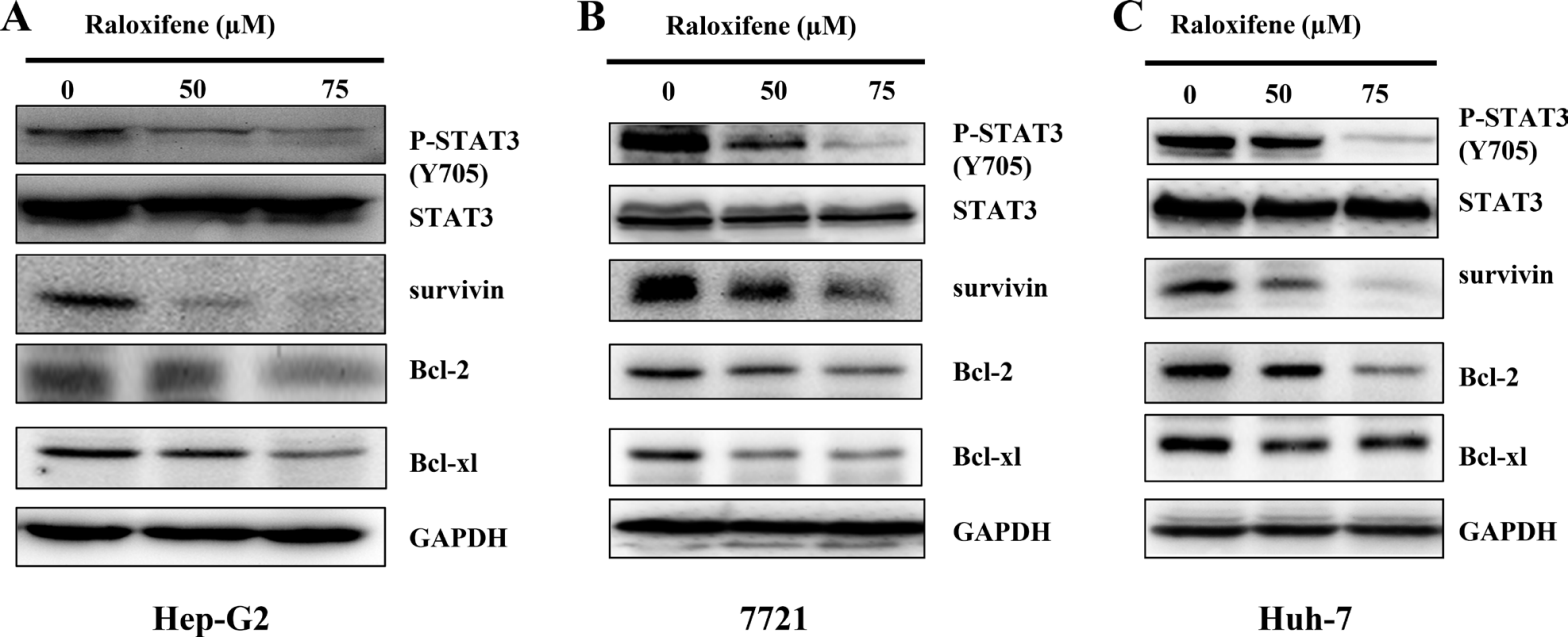
Raloxifene inhibited constitutive STAT3 phosphorylation and the target genes of STAT3 in liver cancer cell lines With the treatment of Raloxifene for 24 h, phosphorylation of STAT3 and the downstream targets of STAT3 were tested by western blot. The results indicated that P-STAT3 was inhibited by Raloxifene in Hep-G2 (**A**), 7721 (**B**) and Huh-7 (**C**) liver cancer cells. The downstream targets of STAT3 were also down regulated by Raloxifene.

To detect the inhibitory effect of Raloxifene on persistent STAT3 phosphorylation, Hep-G2 (Figure 3A), 7721 (Figure 3B) and Huh-7 (Figure 3C) cancer cell-lines, which elevates the levels of STAT3 phosphorylation, were treated with Raloxifene (50, 75 μM) for 24 hours. Western blot analysis showed that Raloxifene inhibited persistent STAT3 phosphorylation. We also examined the expression of STAT3 target genes, such as Bcl-2, Bcl-xl, and survivin in liver cancer cells by western blot. With the treatment of Raloxifene for 24 hours, the expression of Bcl-2, Bcl-xl, and survivin was reduced in Hep-G2 (Figure 3A), 7721 (Figure 3B), and Huh-7 (Figure 3C) liver cancer cell-lines.

### Raloxifene inhibited cell viability and colony forming capacity

**Figure 4:**
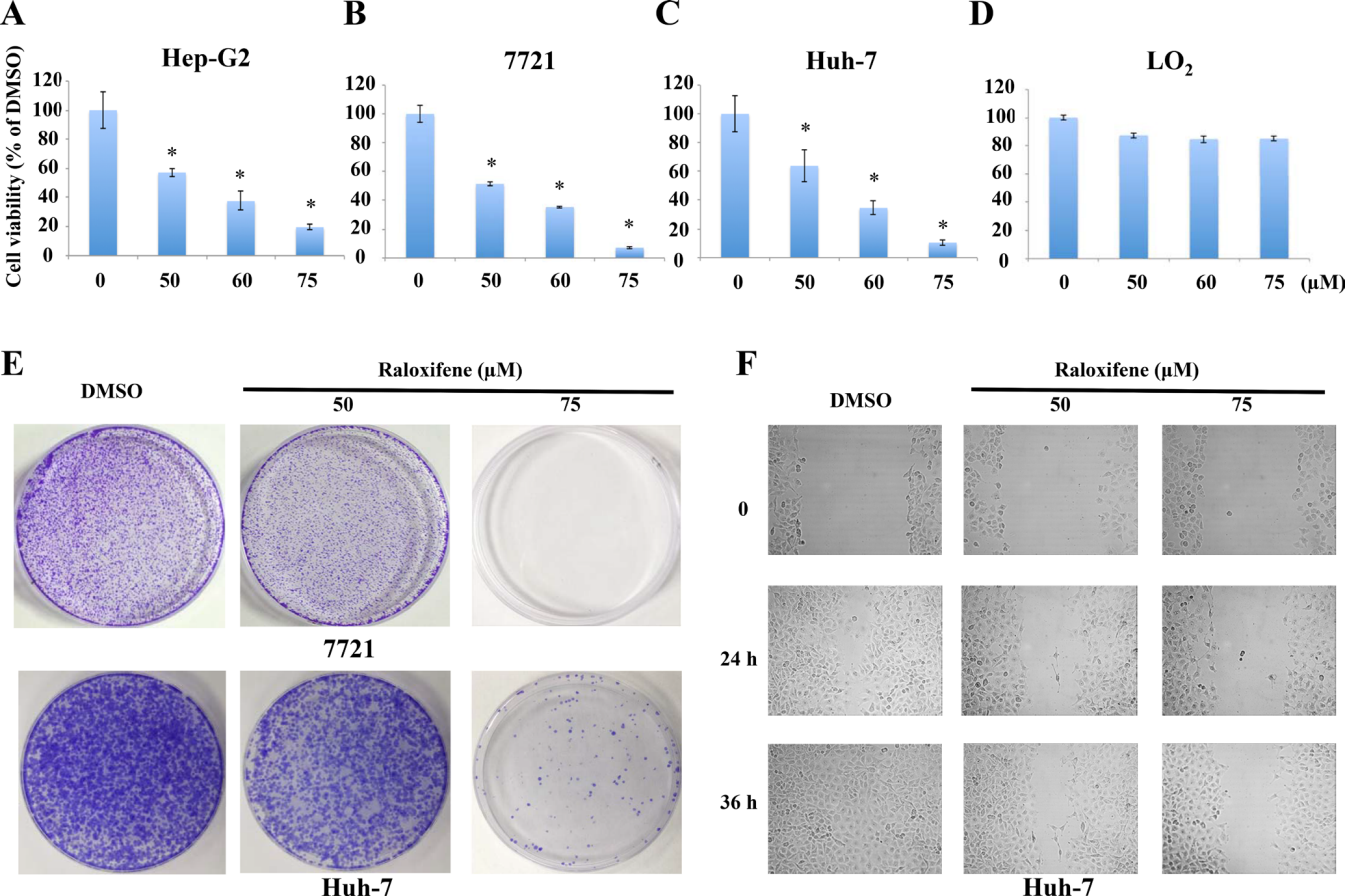
Raloxifene inhibited the viability of liver cancer cells MTT assay showed that the viability of liver cancer cells was inhibited with the treatment of Raloxifene for 24 h in Hep-G2 (**A**), 7721 (**B**), and Huh-7 (**C**). However, Raloxifene did not significantly inhibit the cell viability of LO2, which is a normal hepatocyte cell line (**D**). Raloxifene inhibited colony formation in Huh-7 and 7721 liver cancer cells (**E**). Cells colony formation ability was investigated after treatment with Raloxifene for 4 h. The results showed that Raloxifene suppressed the proliferation and regeneration ability of Huh-7 and 7721 cancer cells. Raloxifene inhibited cell migration in Huh-7 cancer cells (**F**). With Raloxifene treatment for 4 h, wound-healing assay was used to test the migration ability of Huh-7 cancer cells. Raloxifene inhibited cell migration with the concentration of 50 and 75 μM.

As IL-6/GP130/STAT3 signaling is essential for cell viability and colony formation in liver cancer cells, we examined cell viability by MTT assay. Treatment with Raloxifene (50, 60, 75 μM) for 24 hours resulted in a dramatic decrease of cell viability in a dose-dependent manner in Hep-G2 (Figure 4A), 7721 (Figure 4B) and Huh-7 (Figure 4C) cancer cells. The IC50 values for Raloxifene were 50.488 to 53.858 μM in liver cancer cells. In addition, we investigated the effect of Raloxifene on cell viability of LO2 liver cell line, which showed that Raloxifene proved weak inhibition effect on normal liver cells (Figure 4D).

We also examined the efficacy of Raloxifene in inhibiting the proliferation and regeneration potential of cancer cells. The results demonstrated that Raloxifene remarkably inhibited colony forming capacity of 7721 and Huh-7 liver cancer cells (Figure 4E).

### Raloxifene inhibited cell migration in Huh-7 liver cancer cells

STAT3 has been shown to be involved in wound healing and cell migration of cancer cells, which might lead to invasion and metastasis. We evaluated the effect of Raloxifene on cell migration in Huh-7 liver cancer cells by wound healing assay. Our results indicated that Raloxifene could suppress cell migration in a dose-dependent manner (Figure 4F).

### Raloxifene inhibited STAT3 nuclear translocation in liver cancer cells

**Figure 5:**
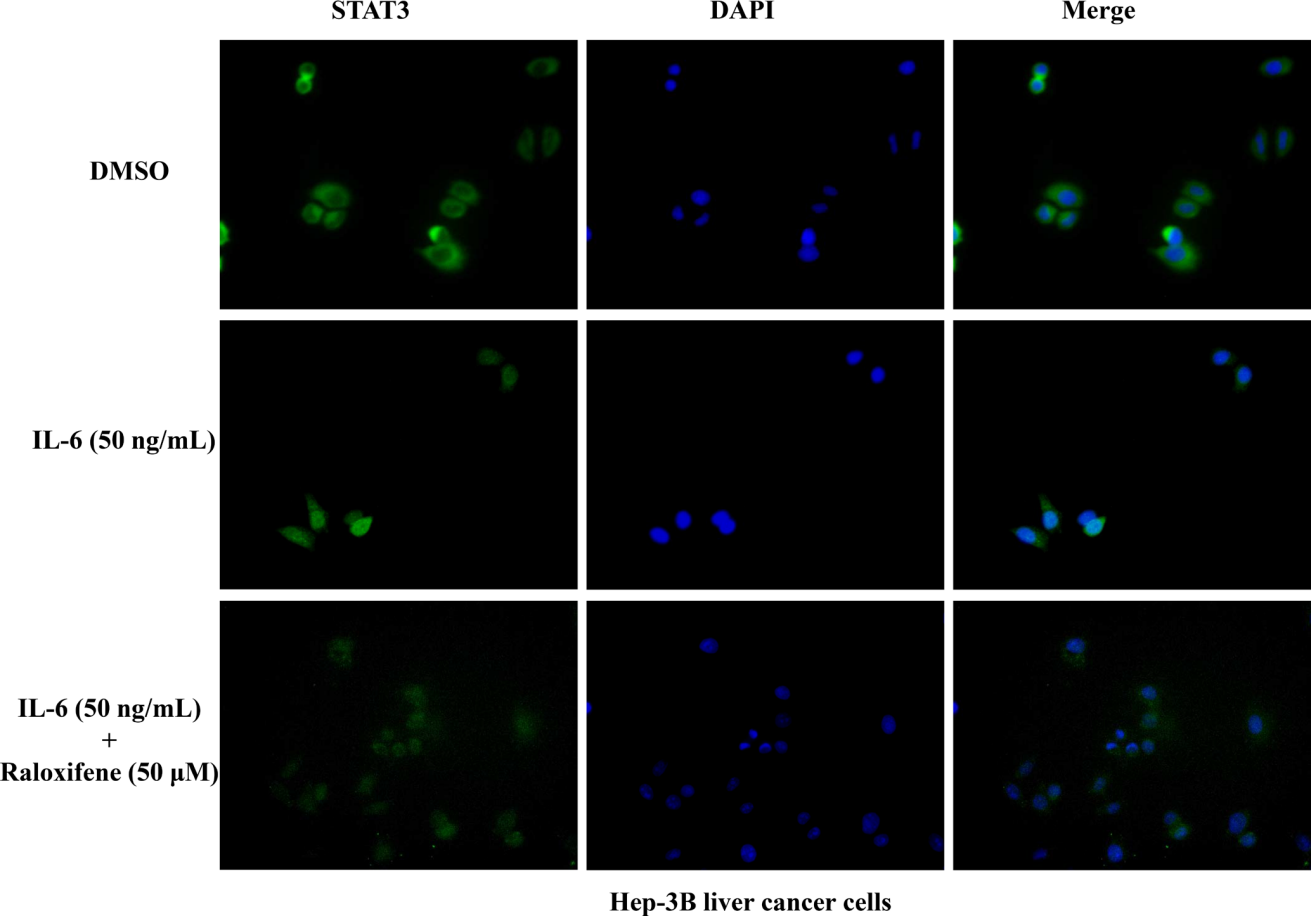
Raloxifene inhibited STAT3 translocation to nucleus induced by IL-6 in Hep-3B cancer cells After serum-free overnight, Hep-3B liver cancer cells were pretreated with Raloxifene for 2 h, followed by IL-6 (50 ng/mL) induced for another 30 min, then processed of STAT3 nuclear translocation detection by immunofluorescence staining.

As a transcription factor, the translocation of STAT3 from cytoplasm to nucleus is very important. So we detected the effect of Raloxifene on STAT3 translocation in Hep-3B liver cancer cells. Cells were cultured in serum-free medium for 8 hours to make STAT3 locate in the cytoplasm. With the pretreatment of Raloxifene for 2 hours, IL-6 was added to induce for 30 min. Then the immunofluorescence staining assay was used to evaluate the translocation of STAT3. Our results showed that STAT3 was phosphorylated and translocated into the nucleus with the induction of 50 ng/ml IL-6. While with the pretreatment of Raloxifene, STAT3 was still located in cytoplasm (Figure 5). These findings demonstrated that STAT3 transcriptional function was impaired via the consequent suppression of STAT3 phosphorylation by Raloxifene in liver cancer cells by blocking nuclear translocation.

### Raloxifene induced cell apoptosis in liver cancer cells

**Figure 6:**
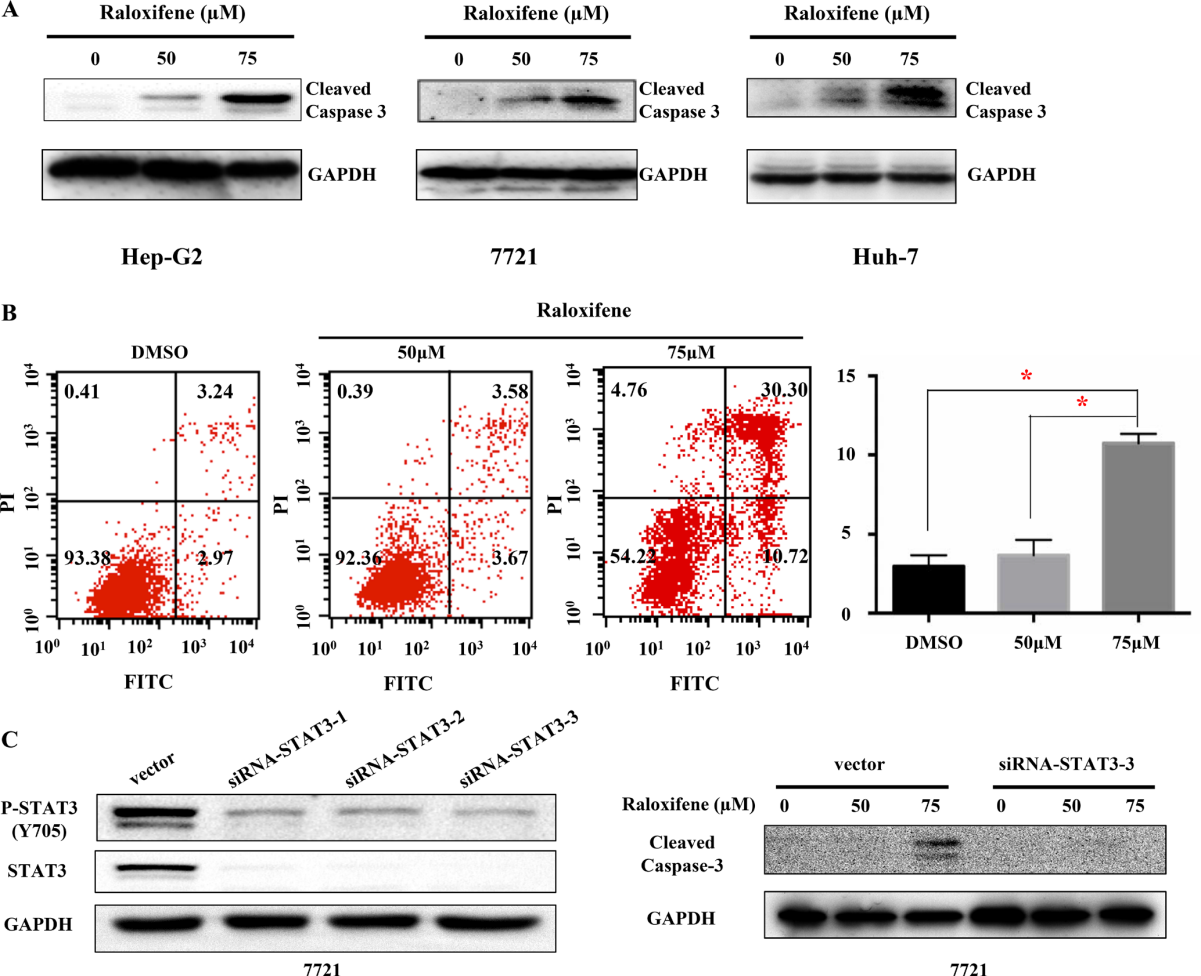
Raloxifene induced cell apoptosis of liver cancer cells With Raloxifene treatment for 24 h, the cleaved caspase-3 was tested by western blot. Raloxifene induced caspase-3 cleavage in Hep-G2, 7721, and Huh-7 (**A**), which indicated cell apoptosis. The Annexin V-FITC staining was used to test the distribution of phosphatidylserine, which is also used to indicate cell apoptosis (**B**). 7721 liver cancer cells was transfected with siRNA-STAT3 to knock down the expression of STAT3, and then the expression of P-STAT3 was also decreased. Raloxifene was added for another 24 hours and cleaved caspase-3 was detected by Western Blot. Raloxifene did not induce the cleavage of Caspase-3 when STAT3 was knocked down by siRNA-STAT3 transfection (**C**).

To examine the effect of Raloxifene on cell apoptosis, cleaved caspase-3 that indicated cell apoptosis was tested after treatment with Raloxifene for 24 hours by western blot in Hep-G2, 7721 and Huh-7 liver cancer cells (Figure 6A). The results showed that Raloxifene induced cell apoptosis by up-regulation of cleaved caspase-3. Further, we investigated the distribution of phosphatidylserine in cell membrane with the treatment of Raloxifene for 6 hours using Annexin V-FITC staining by flow cytometry assay. Our results showed that phospharidylserine turned inside-out with the treatment of Raloxifene (50, 75 μM) in 7721 cancer cells, which indicated cell apoptosis (Figure 6B).

Further more, we explored the effect of Raloxifene on 7721 cancer cells after siRNA-STAT3 transfection. After siRNA-STAT3 transfection, the expression and activation level of STAT3 in 7721 cells were down regulated (Figure 6C). After Raloxifene added for 24 hours, cleaved caspase-3 was detected by western blot assay. Compared with vector group, Raloxifene had no effect on cell apoptosis, which indicated Raloxifene inhibited cell growth by targeting IL-6/GP130/STAT3 signaling (Figure 6C).

### Raloxifene suppressed tumor growth of liver cancer cells in mouse model *in vivo*

**Figure 7:**
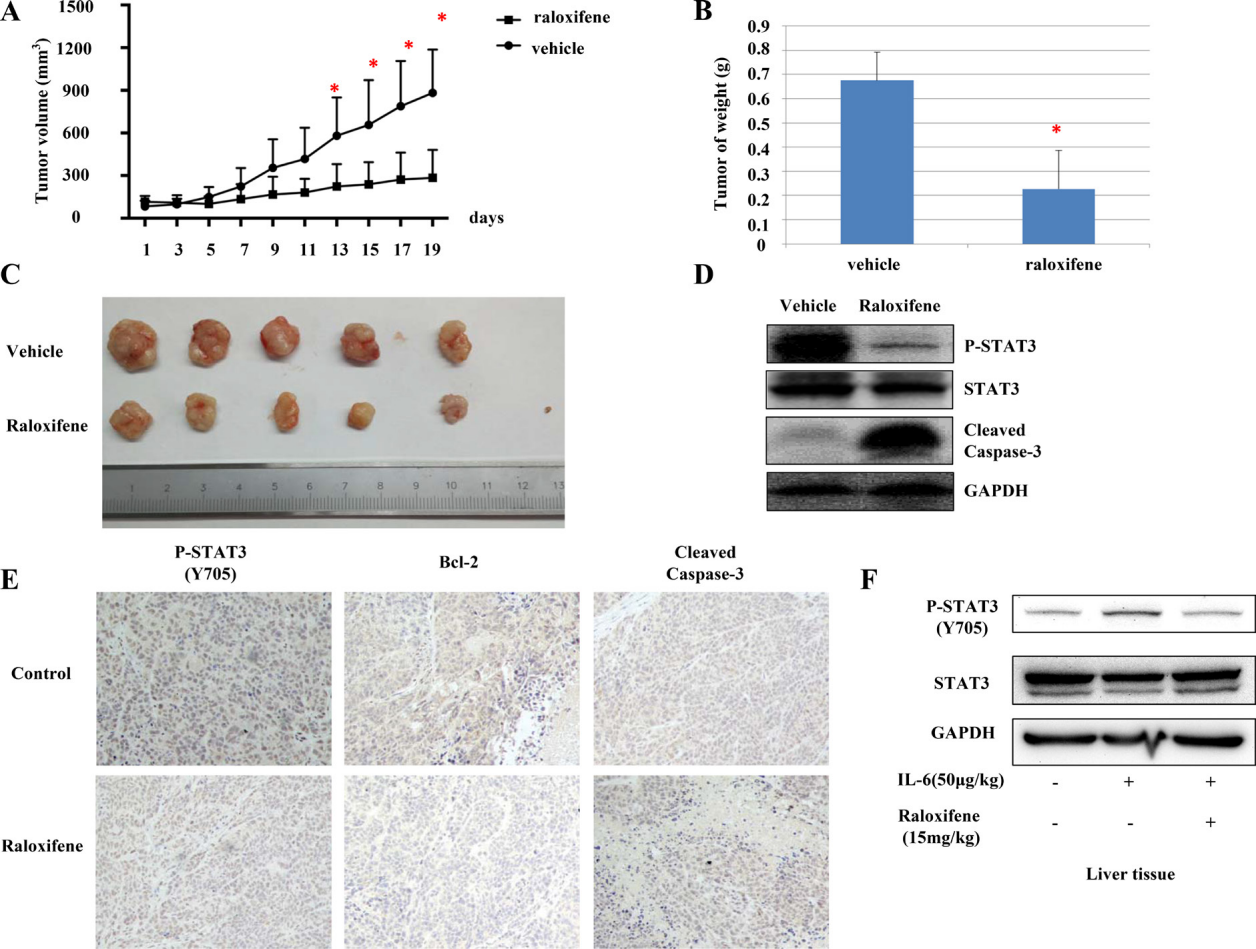
Raloxifene suppressed tumor growth of liver cancer cells *in vivo* Raloxifene decreased cell volume compared with vehicle group. The next day after Hep-G2 cancer cells injected, the mice were randomized to give daily oral dosages of 15 mg/kg of Raloxifene or vehicle control for 19 days and tumor volumes were determined every other day (**A**). After tumors harvested, weighed every tumor by electronic scale (**B**) and determined the volumes of tumors (**C**). Raloxifene inhibited P-STAT3 but not total STAT3, and induced caspase-3 cleavage in mouse xenografts *in vivo* (**D**). Raloxifene also inhibited STAT3 phosphorylation, decreased the expression of Bcl-2 and induced apoptosis as shown by IHC staining (**E**). Raloxifene (15 mg/kg) pretreated for 24 hours and IL-6 injected for another 2 hours in nude mice, then P-STAT3 of liver tissues was detected by western blot (**F**). Raloxifene suppressed the phosphorylation of STAT3 induced by IL-6 in liver tissues of nude mice, which indicated Raloxifene could inhibited IL-6/STAT3 signaling *in vivo* with one dose of 15 mg/kg.

We further tested the suppression effect of Raloxifene on tumor growth in nude mice xenograft models *in vivo*. The mice were randomly divided into two groups. Compared with vehicle-treated controls, the tumor volume (Figure 7A, 7C) and weight (Figure 7B) of mice treated with Raloxifene was significantly decreased. STAT3 phosphorylation of tumor tissue samples from these mice was also decreased by Raloxifene, suggesting inhibition in STAT3 phosphorylation resulting in the suppression of tumor growth in mice. Further, the cleavage of caspase-3 was tested by western blot, and the results showed that Raloxifene induced caspase-3 cleavage in xenograft (Figure 7D). IHC staining showed that Raloxifene inhibited STAT3 phosphorylation and Bcl-2 compared with control group. Further more, Raloxifene induced caspase-3 cleavage in rumor tissues (Figure 7E).

To further evaluate whether Raloxifene could reach an effective concentration *in vivo* with a dose of 15 mg/kg, another experiment was designed. Raloxifene (15 mg/kg) was orally administrated to nude mice for 24 hours pretreatment, and IL-6 (50 μg/kg) was given via peritoneal injection for 2 hours. Phosphorylation level of STAT3 in liver tissues was detected. Raloxifene inhibited STAT3 activation induced by IL-6 (Figure 7F), which induced Raoxifene at 15 mg/kg dose could reach an effective concentration *in vivo*.

## DISCUSSION

It is well known that cytokines not only contribute to hemeostasis via immune responses and biological defense, but they are also involved in cancer, inflammation, allergies, and autoimmune diseases. IL-6 is a major cytokine in inflammation and oncogenesis. It has been reported that excess IL-6 production is closely associated with cancer cachexia [17] and inflammation hepatocellular adenoma [18]. It has been reported that circulating IL-6 is a risk indicator and is strongly correlated with adverse prognosis in hepatocellular carcinoma (HCC). The development of HCC depends on paracrine IL-6 production by inflammatory cells and acquired autocrine IL-6 signaling [19–20]. One of the most important downstream target genes of IL-6 is STAT3, which is constitutively activated in various types of cancer, including liver cancer. STAT3 activation results in the expression of downstream genes that promote cell proliferation and provide resistance to apoptosis, such as cyclin D1, Bcl-2 and Bcl-xl respectively [3, 5, 8, 21]. Consequently, for clinical application, development of a small molecular antagonist targeting to IL-6/GP130 is anticipated because of superiority in therapeutic effect, oral absorbency, and antigenicity.

A lot of novel small molecular inhibitors of STAT3 have been reported to suppress cancer cells and tumor growth, such as LLL12 [22], LY5 [23], XZH-5 [24] and so on. However, to date, the translation of anti-STAT3 therapies into clinical trials has been difficult [25–26]. Further, few of IL-6/GP130 inhibitors are efficient in suppressing tumor growth. The purpose of the present work was to search for new anti-IL-6 therapies that apply to clinical trails.

As we descripted in previous section, Madindoline A (MDL-A) was reported to selectively inhibit GP130, resulting in STAT3 phosphorylation inhibition [15]. However, MDL-A is a natural microbial metabolite, which is no longer available from natural resources, and its binding to GP130 is relatively weak. Until now, there is no any other reported IL-6/IL-6R/GP130 antagonist. With the model of MDL-A, We found that Raloxifene exhibited a new function against IL-6/GP130 protein-protein interface using a novel computational strategy for fragment-based drug design by combining multiple ligand simultaneous docking (MLSD) and drug repositioning in our previous study [16].

Approved by Food Drug Administration (FDA), Raloxifene primarily targets the human estrogen receptors (ER), which involve estrogenic actions on bone but antiestrogenic actions on uterus and breast [27]. They are used in the prevention of osteoporosis. Our previous study showed that Raloxifene selectively inhibits STAT3 phosphorylation induced by IL-6 in the GP130/JAK/STAT3 signaling pathway in pancreatic cancer cell-lines (PANC-1) that express GP130, but not ER [16]. Compared with other IL-6/GP130/STAT3 inhibitors which have not used for clinical therapy, Raloxifene has advantages in terms of oral absorbency, security, and stability. So, in this study, we further investigated whether Raloxifene has the ability to inhibit IL-6/GP130/ STAT3 signaling and suppress tumor growth in liver cancer cells.

Our results showed that Raloxifene inhibited STAT3 phosphorylation induced by IL-6, but not by LIF in Hep-3B liver cancer cells. The reason we chose Hep-3B for this study is that the phosphorylation level of STAT3 in Hep-3B liver cancer cells is low. Further, we tested the effect of Raloxifene on STAT1 phosphorylation induced by IFN-γ and IFN-α and P-STAT6 induced by IL-4. The results showed that Raloxifene did not affect other STATs, which indicated that Raloxifene specifically inhibits IL-6 and GP130 binding. As the downstream of IL-6/GP130, phosphorylation of STAT3, as well as STAT3 downstream target genes, were inhibited by Raloxifene in Hep-G2, Huh-7 and 7721 cancer cell-lines. The inhibitory effects of Raloxifene for STAT3 downstream target genes result in an inhibition of cell viability, the induction of apoptosis, inhibition of colony forming ability and cell migration. Furthermore, Raloxifene demonstrated significant inhibition of tumor growth from Hep-G2 mice tumor xenografts via oral administration, which is more close to physiological serum concentration. The fact that Raloxifene hinder Hep-G2 tumor growth and reduce tumor mass *in vivo* suggests a potential therapeutic application.

Raloxifene is a suitable agent for targeting liver cancer and possibly certain type of cancer cells with constitutively activated STAT3 induced by IL-6, due to its ability to inhibit IL-6/GP130 binding as well as their potent growth suppressive activity both *in vitro* and *in vivo*. Moreover, as an approved drug by FDA, the security and permeability of Raloxifene are superior as compared to other reported novel molecular inhibitors of IL-6/GP130/STAT3, such as MDL-A [15], LLL12 [23], LY5 [24], and XZH-5 [25]. Therefore, Raloxifene is more likely to apply to clinical trails. Further, our study provided experimental basis for the expansion of clinical indications of marketed drug.

## MATERIALS AND METHODS

Investigation has been conducted in accordance with the ethical standards and according to the Declaration of Helsinki and according to national and international guidelines and has been approved by the institutional review board of Tongji Hospital.

### Cell culture

Human liver cancer cell lines (Hep-3B, Hep-G2, Huh-7 and 7721) were purchased from ATCC and LO2 cell line was purchased from China Center for Type Culture Collection in Wuhan. All cell lines were frozen within 2 months of receipt and were resuscitated from early passage liquid nitrogen stocks as needed. All cell lines were routinely inspected microscopically for stable phenotype. These cells were cultured in Dulbecco's modified Eagle's medium/high glucose supplemented with 10% fetal bovine serum (Gibico) and 1% penicillin/streptomycin (Sigma). All cell-lines were stored in humidified 37°C incubator with 5% CO_2_.

### Cell viability assay

MTT cell viability assay kit was purchased from Promoter Biotechnology Ltd. Cells were seeded in a 96-well plates (3,000 per well) in triplicates and then incubated with desired concentrations of Raloxifene (Sigma) at 37°C for 24 hours. MTT (10 μL) was added to each sample and incubated for 4 h. Then, 100 μL N, N-dimethylformamide solubilization solution was added to each well and incubated for 4 h and the absorbance was read at 470 nm.

### Western blot analysis

Cancer cells were treated with different concentrations of Raloxifene (50, 75 μmo/L) or DMSO for 24 h. For interleukin-6 (IL-6), interleukin-4 (IL-4), IFN-γ, IFN-α and LIF treatments, Hep-3B liver cancer cell were pretreated with Raloxifene (50, 75 μmo/L) or DMSO for 2 h and IL-6, IL-4, IFN-γ, IFN-α or LIF (Pepro Tech) were then added for 30 minutes before cells were collected. The collected cells were washed with cold PBS and lysed in a modified RIPA buffer (1% Triton X-100, 1% deoxycholate, 0.1%SDS) containing protease inhibitors (1 mM PMSF) and phosphatase inhibitors (1 mM), then subjected to SDS-PAGE. Primary antibodies (Cell signaling Tech) against phospho-specific STAT3 (Tyrosine 705) (P-STAT3, Y705), STAT1 (Tyrosine 701) (P-STAT1, Y701), STAT6 (Tyrosine 641) (P-STAT6, Y641), ERK1/2 (Threonine 202/Tyrosine 204) (P-ERK, T202/Y204), cleaved caspase-3, STAT3, Bcl-2, Bcl-xl, survivin and GAPDH were used for western blot. Then HRP-conjugated secondary antibodies purchased from Santa Cruz Biotechnology were used. The specific proteins were detected with an enhanced chemiluminescence (ECL) Western Blotting kit according to the manufacturer's instructions.

### Colony formation assay

Huh-7 and 7721 cell-lines were plated as single cells in 6-well plates. Cells were treated with varying concentrations of Raloxifene (50, 75 μM) or DMSO for 4 h. Then viable cells were determined by trypan blue (Promoter Biotechnology Ltd) staining and counted. Five thousand viable cells were seeded in 10 cm plates and continued to grow for two to three weeks. Cells were stained with 0.5% crystal violet (25% methanol) after washed with cold PBS for twice and fixed with cold paraformaldehyde for 15 min. After staining, the plates were rinsed with water and dried.

### Immunofluorescence staining

Cells were seeded in a 6-well plate as single cells and pretreated with Raloxifene for 4 h and then IL-6 was added for another 30 min. Cells were fixed with 4% formaldehyde for 30 min, washed with PBS. 5% BSA and 1% Triton-100 were used for blocking and rupture of membrane for 30 min at room temperature, and then stained with anti-human primary antibody at 4°C overnight. STAT3 antibody (1:100) were used. Cells were incubated with anti-rabbit-FITC secondary antibody (1:500, Jackson ImmunoResearch Laboratories, West Grove, PA) or Alexa Fluor Dye (1:100, Alexa Fluor 594 goat anti-rabbit IgG) anti-rabbit secondary antibody (Molecule Probe, Invitrogen) for 2 h at room temperature shielded from light, and then washed with PBST. Cells were incubated for 5 min at room temperature with DAPI (Vector Laboratories, Burlingame, CA) to stain nuclei, washed twice with PBST, and observed using an inverted fluorescence microscope.

### Wound healing assay

Huh-7 cell-line were plated in a 6-well plate (with three lines at the external bottom of each well) and incubated in a 37°C incubator with 5% CO_2_ overnight to form a confluent monolayer. The monolayers were scratched by a plastic tip vertically to the lines and washed by PBS to remove cell debris. Then the cells were treated with varying concentrations of Raloxifene (50, 75 μM) or DMSO for 4 h. Photos of wound closure were taken at the crossing point of lines and scratches every 12 h by the inverted microscope until the wound treated with DMSO was completely closed. Then the distance of wound closure was measured by Photoshop 8.0 software.

### Transfection

7721 liver cancer cells (3 × 10^5^) were cultured in 6-well plates, and transfection with STAT3 siRNA or empty vector using lipofectamine 2000 following the manufacture's instruction (Invitrogen). The STAT3 siRNA were bought from RIBOBIO Company. Then Raloxifene was added for another 24 hours and cleaved caspase-3 was detected by Western Blot

### Flow cytometry

Hep-G2 cancer cell-lines were seeded in a 6-well plate as single cells and treated with Raloxifene (50, 75 μM) or DMSO for 6 h. After removing the culture medium, the cells were collected and stained using the annexin V-TITC Assay Kit (KeyGen Biotech Co. Ltd) followed by the manufacturer's instructions. At least 1 × 10^4^ cells were analysed with flow cytometry for each sample.

### Mouse xenograft tumor model

Human liver cancer cell-line, Hep-G2 (10^7^ cells in 100 μL of sterile PBS and matrigel), were injected subcutaneously into the right flank region of male athymic nude mice of 4–6-week of age. The next day mice were randomly divided into two groups consisting of 5–6 mice per group, and treated with vehicle control or 15 mg/kg Raloxifene (dissolved in 5% DMSO and 95% hydroxypropyl-beta-cyclodextrin solution of concentration of 20%) via oral administration daily. Tumors were measured with a caliper and the volume were calculated using *V* = π (width^2^ * length)/6, and the weight of nude mice were measured every other day. After the treatment with vehicle or Raloxifene for 19 days, tumors were harvested from euthanized mice, weighed by electronic scale, snap-frozen in liquid nitrogen, and stored in −80°C. A portion of tumor tissues was embedded in Tissue-Tek OCT compound, and stored at −80°C until use for IHC staining. The rest of the tissues were homogenized to examine the expression of STAT3 phosphorylation and cleaved caspase-3 by western blot.

### Statistical analysis

The data were presented as the mean ± SD for at least three independent experiments. Statistical analysis was performed with SPSS software (version 13.0). The significant differences between any of the two groups were evaluated by one-way analysis of ANOVA. Then Least-Significant Difference (LSD) evaluated the difference between two groups. Statistical significance was defined as *P* < 0.05.
